# A Comparative Analysis of the Altered Levels of Human Seminal Plasma Constituents as Contributing Factors in Different Types of Male Infertility

**DOI:** 10.3390/cimb43030093

**Published:** 2021-09-26

**Authors:** Ashutosh Vashisht, Pankaj Kumar Ahluwalia, Gagandeep Kaur Gahlay

**Affiliations:** 1 Department of Molecular Biology and Biochemistry, Guru Nanak Dev University, Amritsar 143005, Punjab, India; ashutosh.mob@gndu.ac.in; 2 Department of Pathology, Medical College of Georgia, Augusta University, Augusta, GA 30912, USA; pahluwalia@augusta.edu

**Keywords:** male infertility, seminal plasma, trace elements, ART, metabolomics, biomarkers

## Abstract

(1) Background: The relationships between the biochemical and immunological components in seminal plasma and their physiological effects on male reproductive system have been underreported. In this study, we evaluated the potential of several seminal plasma biochemical and immunological markers in the pathophysiological developments of the infertile male patients. The study was designed to identify and assess different markers that may be associated with semen functions in different types of male infertility. (2) Methods: A total of 50 infertile male patients who underwent checkup for fertility assessment and 50 fertile controls were included in this study. The complete medical history of each recruited participant was reviewed. The infertile sub-groups (non-obstructive azoospermia (NOA), asthenozoospermia (AS), normozoospermic infertile (NI), and oligozoospermia (OZ)) were characterized based on sperm motility and concentration, while NI patients were included after a thorough check up of their female partners as well. We investigated each sample for 21 different analytes, enzymes, trace elements, and immunological markers to find crucial markers posing as contributing factors to a specific type of male infertility. (3) Results: The levels of 15 out of 21 markers, assayed from the seminal plasma of infertile males, were significantly altered in comparison to fertile controls (*p* < 0.05). For the first time, microprotein levels were also analyzed. The presence of monocytes, lymphocytes, and granulocytes was limited to semen from NOA patients, while a significant increase in the level of platelets was observed in AS. Hierarchical clustering and ROC-AUC analysis identified the three most significant markers (zinc, LDH, and TG) for the healthy control group and asthenozoospermic group (AUC, of 0.92 and 0.81, respectively). (4) Conclusions: The altered levels of biochemical and immunological markers in seminal plasma might be associated with the different male infertility profiles and could be required for the sperm metabolism and maintenance. However, a larger sample size and follow up analysis is required for establishing the hypothesized panel of markers as biomarkers at clinical stage.

## 1. Introduction

Infertility is a widespread health issue that affects approximately 15% of reproductive aged couples globally; approximately half of these cases are related to male infertility [1,2]. Regardless of the many advances in assisted fertilization in recent years, favorable outcomes remain disturbingly low. Semen analysis (spermogram), which evaluates sperm motility, sperm structure, sperm concentration, and biochemical as well as microbiological parameters, is the most widespread, easy, and non-invasive method to understand the etiology of male infertility [3]. Based on sperm motility and concentration, male infertility can be broadly classified as asthenozoospermia (AS: reduced sperm motility), non-obstructive azoospermia (NOA: no sperm), normozoospermic infertile (NI: normal sperm), and oligozoospermia (OZ: reduced number of sperm). However, the current understanding of anomalous sperm function based on spermogram remains limited.

Alternatively, seminal plasma, the liquid component of semen, is coming up as a diagnostic fluid as it is rich in different biochemical as well as immunological markers [4,5]. It consists of secretions from the testis, epididymis, and male accessory glands and provides a medium of transport for the sperm. The components of seminal plasma are also essential in providing nutrition to spermatozoa and help in the regulation of capacitation, sperm motility, semen coagulation, and liquefaction. They also protect the sperm during its passage through the female reproductive tract and play a role in fertilization [6,7]. Hence, perturbations in the seminal plasma components may affect these processes and in turn fertility. Evaluations of these changes may thus be used as signatures for different types of male infertility and to provide an insight to further characterize the reason(s) for the same [8,9,10].

Recently, metabolomic profiling of human seminal plasma using NMR, ultra-performance liquid chromatography-tandem mass spectrometry (UPLC-MS/MS), and real-time quantitative PCR (RT-qPCR), etc. has been performed to explore perturbations in different types of male infertility [9,11,12,13]. All these studies have indicated an imbalance in biochemical parameters amongst different infertile subgroups in comparison to age-controlled healthy fertile males, but the use of expensive techniques and/or the number of biochemical parameters evaluated has become the limiting factor in using these for developing diagnostics. Furthermore, some of these studies either primarily focused on a single infertile category or considered different types of male infertility as one group [13,14,15,16]. Additionally, none of these studies focused on developing a signature panel of markers to distinguish between the different infertile subgroups. Altered profiles for some immunological parameters have also been observed in infertile males [17,18], but their correlation with the overall semen parameters and various biochemical factors is lacking.

To overcome these limitations, the present study was performed to identify seminal plasma components associated with different types of male infertility. Selective markers implicated in sperm motility, capacitation, quality, and number of sperm were identified and analyzed in the seminal plasma of patients with infertility (*n* = 50; OZ, AS, NOA, and NI) after comparison with age-matched fertile males (*n* = 50). Notably, this study investigated and compared the biomarker profile in NOA, OZ, AS, and NI, which has previously not been carried out.

## 2. Materials and Methods

### 2.1. Ethical Consent

Informed written consent was obtained from all participants in the present study prior to the collection of clinical samples and the study was approved by the Institutional Ethics Committee for Human Research, Guru Nanak Dev University, Amritsar (EC NO.576/HG/29/03/2018). All seminal plasma samples were collected from Satjot Human Reproduction Hospital and Research Centre, Amritsar, Punjab, India.

### 2.2. Sample Collection

A total of 749 individuals were screened for eligibility during the period of September 2018 to September 2020, who visited the fertility hospital for either intracytoplasmic sperm injection (ICSI) or in vitro fertilization (IVF) treatment. From 749 individuals, 50 met the inclusion criteria that included clinically diagnosed fertility complications for over 1 year. The exclusion criteria included men with chronic diseases, urogenital infections, varicocele, alcohol consumers, smokers, and those with diseases that could lead to immotile/abnormal sperm or azoospermia. Seminal plasma was obtained from 50 infertile patients (OZ, AS, NOA, and NI) and 50 age-matched healthy fertile controls. The subjects included in this study belonged to the age group of 24 to 40 years and did not self-report any other disorders. Rigorous inclusion and exclusion criteria were employed to study the patient groups. In the NOA study group, only non-obstructive azoospermia patients were included. For NI study group, patients with normal sperm parameters and normal female partners—i.e., females with normal follicular stimulating hormone (FSH) and luteinizing hormone (LH) levels, normal ovulation, and tubal patency—were included for the analysis. Semen specimens were collected through masturbation after 5–7 days of sexual abstinence and incubated at 37 °C for liquefaction, followed by routine semen analysis as per World Health Organization (WHO) guidelines [3]. These included the sperm count, sperm motility, viability, and sperm concentration. The remaining semen sample was centrifuged at 12,000× *g* for 5 min and the upper layer of seminal plasma was analyzed for biochemical as well as immunological markers. All semen samples which did not undergo liquefaction and with a volume less than 2 mL were excluded to avoid the potential influence on the accuracy of sampling and to ensure that all biochemical and immunological markers were detected. All the samples were analyzed blind.

### 2.3. Biochemical and Immunological Analysis

A total of 21 different biochemical and immunological markers including analytes, enzymes, and trace elements were tested from the seminal plasma of fertile and infertile patients. Glucose (GLC), cholesterol (CHOL), triglycerides (TG), total protein (TP), urea, creatinine (CREAT), albumin (ALB), microprotein (miP), serum glutamic pyruvic transaminase (SGPT), serum glutamic-oxaloacetic transaminase (SGOT) and uric acid (UA) were estimated using commercially available kits from Erba Mannheim, Germany. Creatine kinase (CK) and lactate dehydrogenase (LDH) was analyzed using kits from Coral Clinical Systems, India. The biochemical markers in the seminal plasma samples were examined using the Clinical Chemistry Analyser–C71 (VWR, Radnor, PA, USA). Calibration and quality control products were provided by the respective companies. For zinc estimation, seminal plasma (0.5 mL) was mineralized by placing the samples in mineralization tubes and adding 1 mL of a nitric and hydrochloric acid mixture (HNO_3_-HCl; 4:1), and heating at 120 °C for 65 min. The resulting solution was diluted to 10 mL with demineralized water and the digested samples were analyzed for zinc concentration using Agilent 240 FS AA Atomic absorption spectrometer (Agilent Technologies, Santa Clara, CA, USA). Calcium (Ca^2+^) and sodium (Na^+^) ions were detected via Roche 9180 Electrolyte analyser (Roche Diagnostics, Mannheim, Germany). White blood cells (WBCs), monocytes (MON), lymphocytes (LYM), granulocytes (GRAN), and platelets (PLT) were analyzed using ABX Micros ES 60 analyser (Horiba Medicals, Kyoto, Japan). The calibration and examination of quality control products for all biochemical and immunological markers were performed according to the manufacturers’ protocols.

### 2.4. Metabolome Analysis

To analyze the metabolic pathways impacted (if any) due to altered levels of seminal plasma glucose, cholesterol, zinc, and sodium, MetaboAnalyst 4.0 was used [19]. It uses high-quality KEGG metabolic pathways as the knowledge base and well-established algorithms for pathway analysis. The analyte data were input in the pathway analysis module as a csv list of compounds. The fertile control was designated as 0 and infertile patient as 1. Only exact compound matches were taken and the ones without a match were excluded from the subsequent pathway analysis. A global test algorithm was used for pathway enrichment analysis, which evaluated if the observed analytes in a pathway appeared more frequently than expected by random chance in a given dataset. The relative-betweenness centrality algorithm was used for pathway topology analysis, which evaluated the potential importance of a given analyte based on its position within a pathway. The *Homo sapiens* KEGG pathway library was used for this analysis and a *p* value of ≤0.05 was taken to be significant for both pathway enrichment as well as topology analysis.

### 2.5. Statistical Analysis

The data obtained were saved in Microsoft Excel and was analyzed using GraphPad Prism 8 (version 8.0.2, San Diego, CA, USA) and JMP-Pro (version 14.0.0, SAS Institute, Cary, NC, USA). The values for the various biochemical and immunological markers in the seminal plasma of fertile and infertile patients are presented as Mean ± SD and were compared by ANCOVA with age as a covariate. Post hoc (Bonferroni) analysis was utilized for ANCOVA comparison across groups. The box and whisker plot captured the differences between subgroups. The line through the middle of the boxes corresponds to the median and the lower and the upper lines correspond to interquartile range (IQR) with 25th and 75th percentile, respectively. The whiskers captured the variability outside the IQR. To identify deviations in normal healthy parameters which when perturbed lead to an unhealthy diseased state, we partitioned the data into two groups: fertile and infertile. To visualize the overall variations in the biomarkers, a volcano plot was generated to identify significant clinico-pathological variables. For this, an unpaired *t*-test per variable was performed between the fertile and infertile group and the results were plotted as a volcano plot. Each parameter was tested individually without assuming a consistent SD and the statistical significance was set at 0.05. Hierarchical clustering (estimated via cluster analysis based on Ward’s algorithm), displayed as a dendrogram and constellation plot, was used to identify clusters with similar expression. To further identify the most significant variables, logistic procedures were performed on all the markers through JMP-Pro using a previously published methodology [20]. Univariate receiver operating characteristics (ROC) analysis was performed to quantify the classification power of individual biomarkers. After univariate ROC analysis, the 3 most significant markers were combined to generate a 3-marker panel through multivariate ROC analysis. The ROC curve was analyzed for different subgroups and was plotted as sensitivity (true positives) against 1-specificity (false positives). For all statistical analyses, a *p* value of <0.05 was taken to be significant.

## 3. Results

### 3.1. Analysis of Semen Parameters among the Fertile and Infertile Groups

Three parameters, namely age, sperm count, and sperm motility, were analyzed among the fertile controls (FC) (*n* = 50) and patients with AS (*n* = 13), NOA (*n* = 16), NI (*n* = 10) and OZ (*n* = 11) (Table 1).

One-way ANOVA suggested a significant difference between the mean age, sperm count, and sperm motility of FC vs the other infertile subgroups.

### 3.2. Assessment of Various Biochemical and Immunological Markers between the Fertile and Infertile Subgroups

Thereafter, the levels of a total of 21 different biochemical (including nine analytes, four enzymes, and three trace elements) and five immunological markers (Mean ± SD) were assessed in the seminal plasma of fertile and infertile groups (Table 2). Of the 21 markers which were investigated in this study, 15 were found to be significantly (*p* < 0.05) altered as compared to the fertile control (Table 2). UA, Urea, TP, ALB, CREAT and CAL were the six markers in which no significant change was observed.

### 3.3. Assessment of Altered Metabolites in Infertile Subgroups and Their Effect on Metabolic Pathways

**Figure 1:**
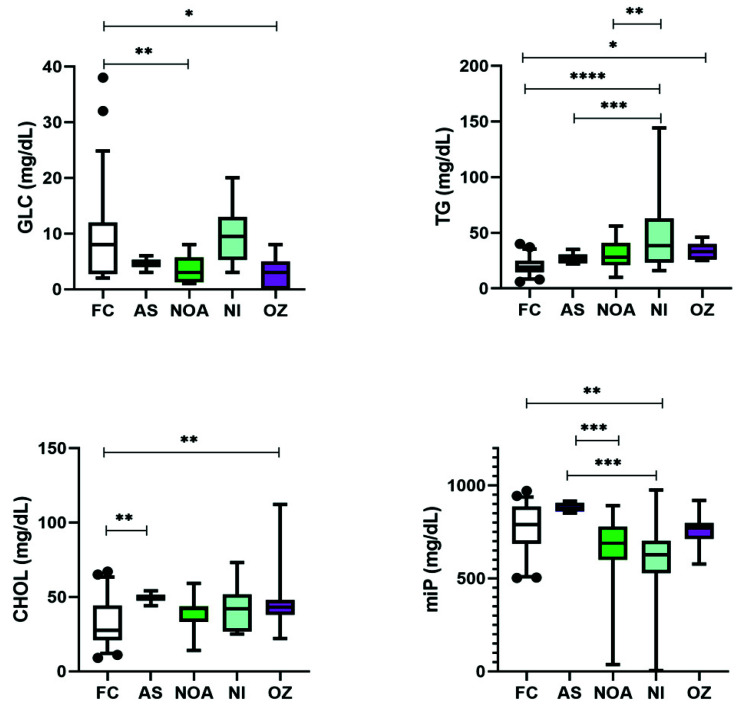
Box and whisker plots depicting the levels of various analytes in infertile patients. Middle line: median; boxes: interquartile range; whiskers: extremes; points: outliers. * Significantly different, *p* = 0.01 to 0.05. ** Significantly different, *p* = 0.001 to 0.01. *** Significantly different, *p* < 0.001. **** Significantly different, *p* < 0.0001.

Four (GLC, TG, CHOL, and miP) out of the nine analytes studied were found to be significantly altered in the infertile patients (Table 2). As depicted in Figure 1, GLC was significantly lower in NOA and OZ as compared to the FC. However, no significant difference in the GLC levels was observed within the different infertile subgroups. TG levels of NI and OZ patients were significantly higher as compared to FC while within the infertile subgroups significant difference in the altered levels was observed only between NI and AS or NI and NOA. When comparing CHOL levels, only AS and OZ had significantly higher levels than FC, and like GLC, no significant difference was observed between the infertile subgroups. With miP, only NI had significantly lower levels than FC. Although AS patients had higher miP levels, they were significantly different only from those of NOA and NI patients.

**Figure 2:**
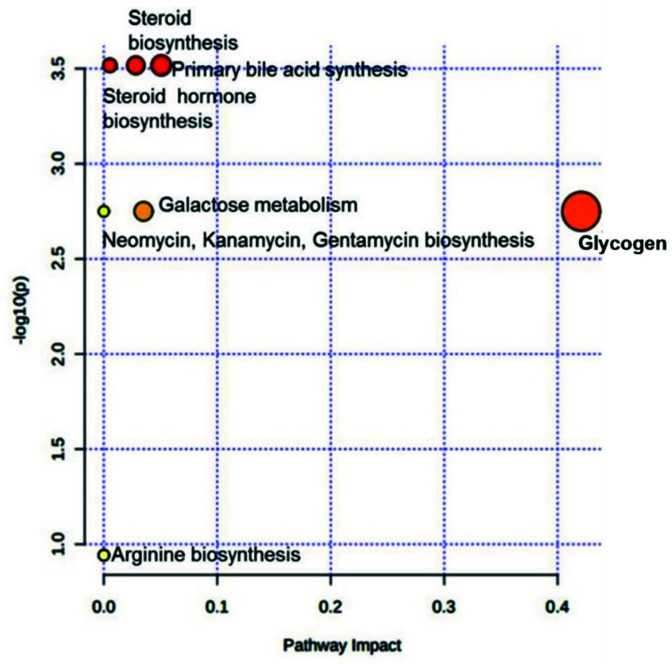
Pathway impact graph depicting analyzed analytes as -log10(p) value. The varying color from yellow to red depicts the number of analyzed compounds in the concerned pathway (red means the pathway consists of a higher number of analytes and light yellow depicts the least number of analytes). Higher significant pathways are depicted away from the Y-axis and greater the impact of an analyte farther away it is observed on the X-axis.

These four analytes, whose levels were significantly altered as compared to the FC, were then analyzed for their effect on various metabolic pathways using MetaboAnalyst 4.0 (Supplementary Table S1). The results indicated that altered levels of these analytes could possibly impact the primary bile acid biosynthesis, steroid biosynthesis, steroid hormone biosynthesis, glycogen metabolism, galactose metabolism, purine metabolism, and arginine biosynthesis (Figure 2). Glucose, which a central part of glycogen metabolism, seems to have severely impacted the pathway (0.42) (Table 3).

### 3.4. Enzymatic Levels Were Altered in Some Infertile Subgroups

**Figure 3:**
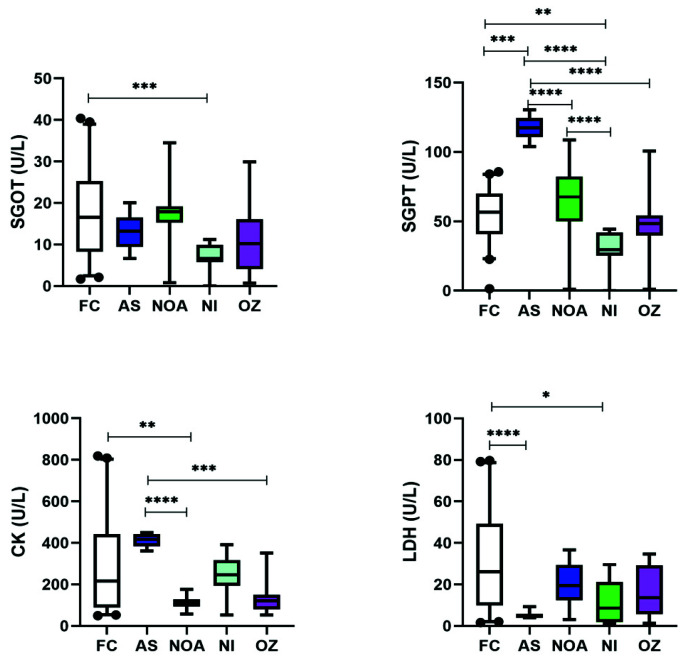
Box and whisker plots depicting the levels of various enzymes in infertile patients. Middle line: median; boxes: interquartile range; whiskers: extremes; points: outliers. * Significantly different, *p* = 0.01 to 0.05. ** Significantly different, *p* = 0.001 to 0.01. *** Significantly different, *p* < 0.001. **** Significantly different, *p* < 0.0001.

The levels of enzymes investigated in this study were also found to be perturbed in the various infertile subgroups (Figure 3). SGOT levels were significantly decreased in NI as compared to FC, but no significant variation was observed between AZ, NOA, NI, and OZ subgroups. On the other hand, the levels of SGPT were found to be significantly increased in AS and NI as compared to FC. AS patients had significantly higher levels of SGPT as compared to NOA, NI, and OZ; and NOA was higher than NI. CK was found to be significantly decreased only in the NOA patients when compared to FC. However, the levels in AS patients were significantly higher than NOA and OZ. LDH levels were significantly lower than FC only in AS and NI. No significant variation within infertile subgroups was observed.

### 3.5. Varying Levels of Zinc and Sodium Were Found among Trace Elements in Infertile Subgroups

**Figure 4:**
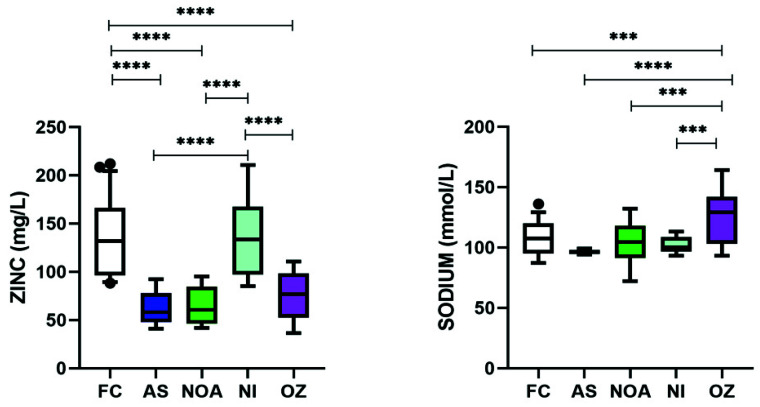
Box and whisker plots depicting the levels of various trace elements and (d) immunological markers in infertile patients. Middle line: median; boxes: interquartile range; whiskers: extremes; points: outliers. *** Significantly different, *p* < 0.001. **** Significantly different, *p* < 0.0001.

Out of the three trace elements investigated, only zinc and sodium were significantly altered (Table 2). Interestingly, zinc levels of NI and FC were comparable (Figure 4 and Table 2). However, the levels in AS, NOA, and OZ patients were significantly decreased when compared to either FC or NI. On the other hand, only OZ patients had significantly increased levels of sodium when compared to either FC or within the different infertile subgroups.

### 3.6. Immunological Perturbations Were Found Only in NOA Patients

**Figure 5:**
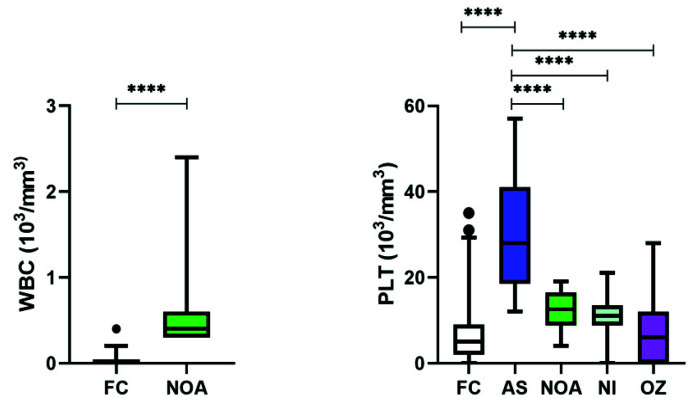
Box and whisker plots depicting the levels of various trace elements and (d) immunological markers in infertile patients. Only platelets and WBCs are shown in the immunological markers as other markers of this category were detected only in non-obstructive azoospermia. Middle line: median; boxes: interquartile range; whiskers: extremes; points: outliers. **** Significantly different, *p* < 0.0001.

Amongst the immunological markers, significant levels of WBCs including LYM, MON, and GRA were detected only in NOA patients (Figure 5 and Table 2). Although PLT was present in the fertile as well as infertile group but only AS patients had significantly higher levels as compared to FC as well as among the different infertile subgroups.

### 3.7. Each Infertile Subgroup has a Characteristic Panel of Markers That Are Altered as Compared to Fertile Controls

When compared with the levels observed in fertile males, altered levels of five markers were seen in AS (CHOL, SGPT, LDH, zinc, and PLT), NI (TG, miP, SGPT, SGOT, and LDH) and OZ (GLC, TG, CHOL, zinc, and sodium) patients, while four were seen in NOA (GLC, CK, zinc, and WBC) (Table 4). Interestingly, miP appears to be a specific marker for NI; CK and WBC for NOA; PLT for AS; and sodium for OZ patients.

### 3.8. Correlation between Sperm Parameters and Various Biochemical and Immunological Markers with Fertility

**Figure 6:**
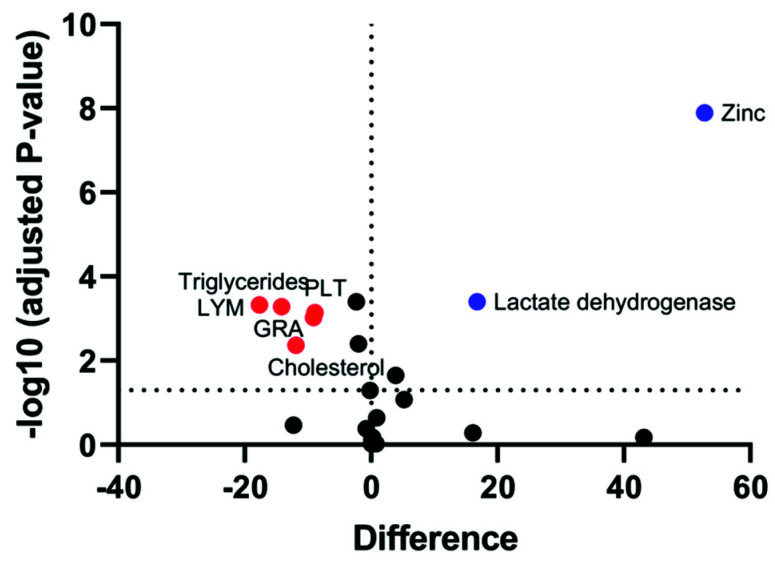
Volcano plot comparing the different biochemical and immunological markers between the fertile control group and the infertile group. An unpaired *t*-test graph of variables was used to differentiate the significantly elevated marker in both groups. The red and blue colors were assigned to significant variables in the infertile and fertile control, respectively, and log10 fold change was observed in the variables. The statistical significance was set at *p* < 0.05. The black dots represent markers that are below the threshold and close to 0 and thus represent non-significant fold change. The values that are farthest from the center were considered as most significant.

To identify the parameters that, when altered, can lead to infertility, different biochemical and immunological markers were compared by a Volcano plot. The Volcano plot distinctly indicated that the fertile control had significantly higher levels of zinc and LDH as compared to the infertile group, with maximum alterations being observed in zinc levels (Figure 6). On the other hand, TG, CHOL, PLT, LYM, and Gran were found to be significantly altered in the infertile group.

### 3.9. Heat Maps Depict Altered Correlation between Various Parameters in the Infertile Group

**Figure 7:**
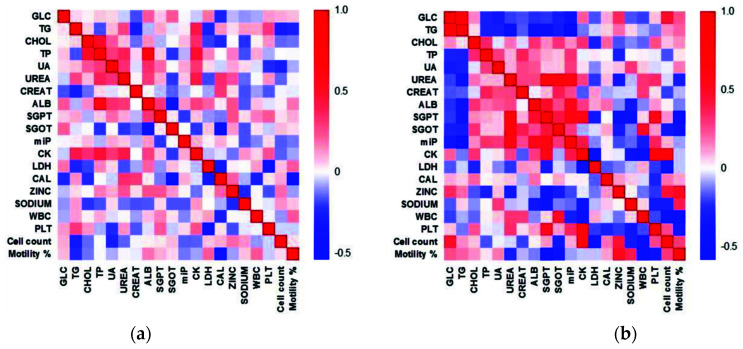
Heat maps depicting the correlation between the biochemical markers, immunological markers, and sperm parameters within the (**a**) fertile control and (**b**) infertile group. The side color bar represents the correlation (r) value from 1 (positive correlation) to −0.5 (negative correlation). Strong red and blue colors depict the highly positive and negative correlated markers, respectively, whereas light red/blue and white represent the components with lesser or no correlation.

The heat maps (shown in Figure 7) depict the correlation analysis between the various biochemical and immunological markers, and the sperm parameters (motility and cell count) depicted changes in infertile patients as compared to fertile controls (Figure 7). Detailed investigations suggested significant (*p* < 0.05) positive correlations between ALB-TP, TP-CHOL, CK-TP, CK-Urea, CK-TG, and Urea-TP in FC (Table 5).

However, a weak negative correlation was seen between CAL-SGOT and surprisingly between CAL and motility. In the infertile group, the strongest correlation was observed amongst the immunological markers. The cell count and sperm motility were negatively affected by the presence of MON, LYM, and GRA (Table 5). The zinc levels had a strong positive correlation with motility (r = 0.5219, *p* < 0.0001), while the SGPT levels negatively affected motility (r = −0.5530, *p* < 0.0001) among the infertile. Significant positive correlation was observed between the cell count and CK levels (r = −0.6554, *p* < 0.0001).

### 3.10. Hierarchical Clustering Defines Two Distinct Groups among the Infertile

**Figure 8:**
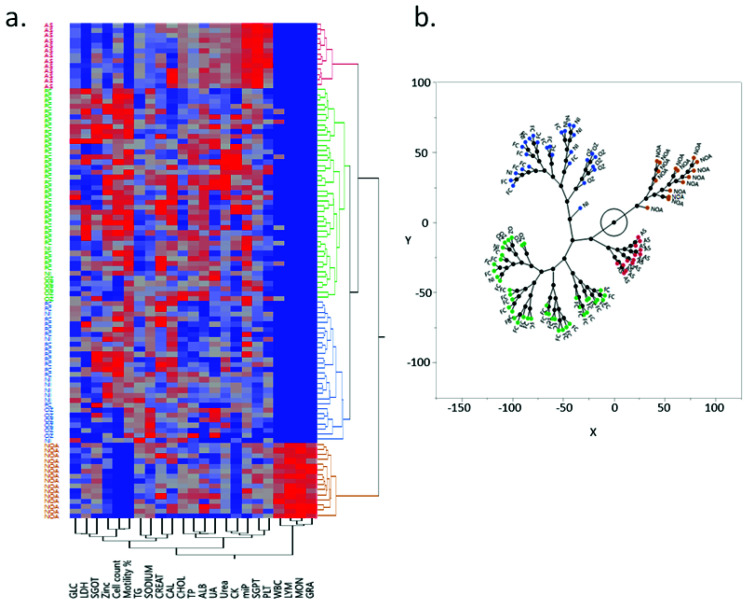
(**a**) Heat map of hierarchical clustering using dendrogram and constellation plot. Hierarchical clustering grouped the study groups into 2 main clusters, which were further divided into sub-clusters. Dendrogram analyses of infertile and fertile groups showed that fertile controls and normozoospermic infertile patients were most closely related, with normozoospermic infertile patients slightly less related to oligozoospermia and control being even less similar to oligozoospermia. (**b**) Constellation plot depicted that AS and NOA patient group profiles were altogether different from each other as well as from other study groups. Both the study groups formed distinct clusters as compared to the rest of the groups.

Correlation data were also used to analyze the relatedness between the different study groups using hierarchical clustering. Hierarchal cluster analysis based on Ward’s method revealed four subgroups based on the scree plot. One of the major subgroups identified was NOA, which had a distinct seminal plasma profile. Among other clusters, AS was another distinct cluster, while other sub-groups consisted of a mix of control and other infertile individuals having similar biochemical and immunological profiles (Figure 8a). The seminal profile of the fertile control was found to be most closely related to NI patients.

**Figure 9:**
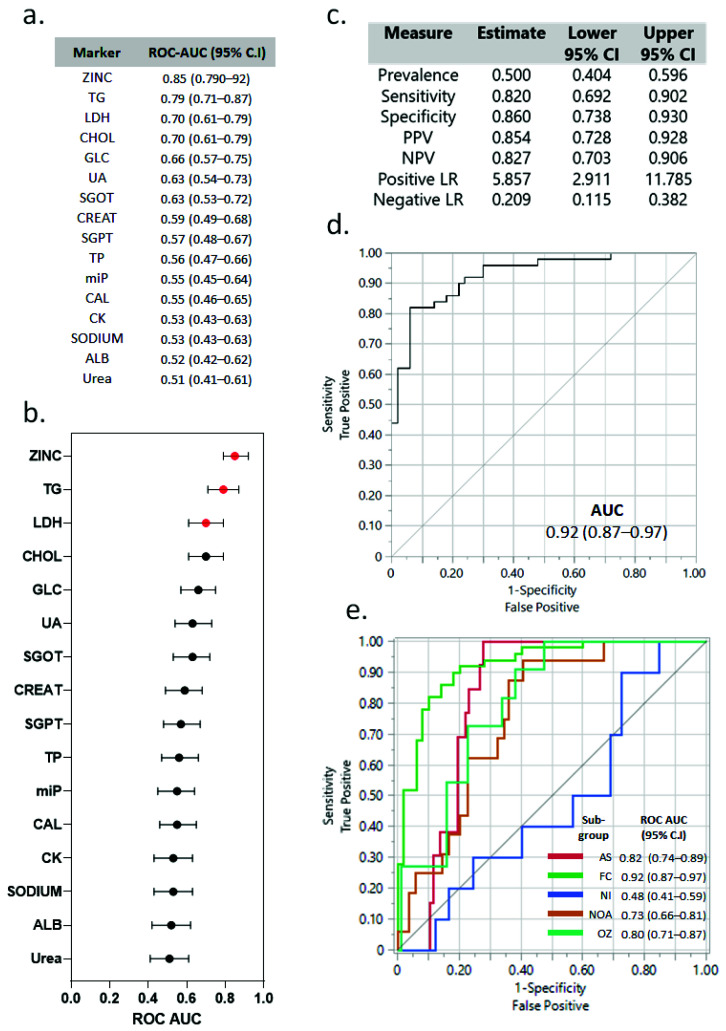
(**a**) Univariate ROC-AUC analysis of biomarkers differentiating fertile from infertile individuals (with 95% CI). (**b**) Forest plot of ROC-AUC of individual markers. (**c**) The predictive power of 3-marker panel (Zn, TG, and LDH). (**d**) Classification power of 3-marker. (**e**) The combined 3-marker (zinc, LDH, and TG) classification of infertility subtypes from fertile control. PPV: positive predictive value; NPV: negative predictive value; positive LR: positive likelihood ratio; negative LR: negative likelihood ratio; FC: fertile control; AS: asthenozoospermia; NOA: non-obstructive azoospermia; NI: normozoospermic infertile; OZ: oligozoospermia.

The constellation plot of seminal plasma profile of fertile control and the infertile group showed that the fertile controls, NI, and OZ patients can be distributed in two clusters (blue and green cluster; Figure 8b). However, NOA (brown) and AS (red) are divided into distinct clusters as compared to the other groups. These findings highlight the complexity of metabolic dynamics and their relatedness to the patient profiles. Furthermore, the results of univariate ROC analysis are depicted in Figure 9a,b. Zinc showed the highest AUC (area under the curve) at 0.85 (0.79–0.92). The other markers with acceptable AUC were TG, LDH, and CHOL with AUCs of 0.79 (0.71–0.87) and 0.70 (0.61–0.79) each. The markers with the poorest discriminatory power were CK, sodium, Alb, and urea, with AUCs of 0.53, 0.53, 0.52, and 0.51, respectively. The combined logistic model of three markers (zinc, LDH, and TG) differentiated fertile from infertile with higher sensitivity, specificity, positive predictive value, and negative predictive value (Figure 9c). Multivariate ROC analysis accurately differentiated fertile from infertile in ROC analysis (ROC-AUC of 0.92 (0.97–0.97); Figure 9d). The subgroup analysis showed the highest predictive ability for the control and AS group (AUC of 0.92 and 0.81, respectively; Figure 8d). Furthermore, the AUC values for NOA and OZ were 0.76 and 0.80, respectively. The AUC was poor for NI (AUC of 0.48).

## 4. Discussion

Spermogram remains the center of infertility evaluation as it provides information on the status of sperm integrity. However, knowledge about the altered levels of various biochemical and immunological markers may improve this evaluation and also increase the efficiency of ART. Keeping this in view, 21 different seminal plasma biomarker levels were evaluated in four different male infertility profiles. Within the analytes, glucose, TG, CHOL, UA, urea, and miP were examined. Glucose is needed at the time of capacitation and is important for sperm motility and its deprivation can affect spermatogenesis by an increase in oxidative stress and cellular apoptosis [21,22,23,24]. In our study, glucose levels were reduced significantly in NOA and OZ patients. This seems to be in concurrence with the MetaboAnalyst analysis, where we found the glycogen metabolism pathways to be significantly impacted due to the presence of glucose in them.

The lipid seminal profile is also equally important for sperm development. In the literature, elevated TG and CHOL levels have been associated with decreased sperm motility, steroidogenesis, and decreased fertility [16,25,26,27,28]. Altered lipid levels can cause oxidative stress, which affects sperm quality and quantity [29]. We also observed significantly elevated TG levels in OZ and NI patients and CHOL in AS and OZ patients.

Interestingly, our study detected decreased levels of miPs specific to NI patients. miPs are single-domain proteins that disrupt protein complexes and have been implicated for the first time in male infertility [30]. While several miPs have been studied in cancer [31], the exact mechanism by which these work in male infertility is yet to be determined.

Apart from analytes, the role of enzymes such as CK, SGOT, SGPT, and LDH was also evaluated. CK is crucial for the generation of adenosine triphosphate (ATP) in human spermatozoa via the chemical shuttle between creatine and creatine phosphate [32,33]. Since significantly altered levels of CK are only observed in NOA, they may affect sperm viability because of reduced ATP levels. Similarly, of the two isoforms of LDH, LDH-type A (LDHA) and type C (LDHC; most abundant), which are present in sperm [34,35], LDHC is also responsible for ATP production [36]. Sperm from LDHC KO mice have decreased progressive and hyperactivated motility, which can be attributed to reduced ATP levels [37]. Thus, low LDH levels in AS and NI patients may affect sperm motility. On the other hand, the other two enzymes SGPT and SGOT in seminal plasma are related to the secretory activity of male accessory sex glands. Our studies found a negative correlation between SGPT and the percentage motility, which, along with low LDH levels, could explain the motility defect in AS sperm. However, increased SGPT levels in AS sperm, which we observed, is in contrast to a recent study wherein low SGPT levels were seen in AS patients and a significant positive correlation existed between SGPT and motility [38].

Besides analytes and enzymes, trace elements were also investigated as these act as cofactors and are essential for spermatogenesis by maintaining oxidative stress levels [39,40]. In our study, levels of three trace elements—sodium, zinc, and calcium—were examined. Out of these, only zinc and sodium were observed to be significantly altered. Low levels of zinc were observed in AS, NOA, and OZ, and a positive correlation between zinc levels and sperm motility was also observed. Zinc is present at high concentrations in healthy seminal fluid and may play a multifaceted role in sperm development. It affects the stability of sperm chromatin [41], can play a regulatory role in the process of capacitation and acrosome reaction via epidermal growth factor receptor (EGFR) activation [42], and influences the semen volume, sperm morphology, and sperm motility, as well as spermatogenesis [43,44,45]. Unlike zinc, sodium is required to maintain intracellular pH in sperm along with H^+^ [46]. The semen pH has been shown to regulate sperm motility, viability, and capacitation through various ion channels [47,48,49]. Significantly elevated levels of sodium, as observed in OZ patients, could be caused due to a defect in these.

Generally, WBCs are absent from the seminal plasma of fertile males, and higher levels of WBCs (leukocytospermia) in infertile patients have been linked with oxidative stress [50,51]. However, so far, no study has associated them with a specific type of infertility. In this study, we observed elevated levels of WBCs (MON, LYM, and GRA) in NOA patients alone, while no WBCs were observed in NI, AS, and OZ. A negative correlation of sperm count and motility with MON, LYM, and GRA was also observed. Our results are consistent with those of another study that also found a negative correlation between leukocyte count and sperm motility [52]. The other immunological factor, PLT, was detected in both fertile and infertile groups, though it was significantly elevated in AS patients. High levels of PLT have been shown to cause deleterious effects on sperm motility [53]. This, along with other factors, may be responsible for reduced sperm motility in AS.

The constellation plot identified the presence of diversity in seminal plasma profiles within the studied groups. The cluster analysis separated the NOA and AS biochemically distinct profiles. A plausible explanation for this could be the role of immunological markers in defining the NOA and AS characteristics in these infertile patients. Additionally, we found that, among the 21 quantified biomarkers, a combination of three markers (Zn, LDH, and TG) showed significantly higher ROC-AUC in discriminating healthy controls and the asthenozoospermic group. The combined three-marker panel should be explored further in different populations for validation.

In this study, we also observed that altered levels of certain seminal plasma components were unique to a particular male infertility profile. Based on these results, a unique panel of markers/a specific marker for each type of male infertility has been hypothesized which can probably be used for the detection of these without the use of expensive technologies or invasive techniques (Table 4). However, this needs to be further investigated before it can be clinically used. The development of such a panel of markers will be particularly useful for the diagnosis of NI patients where the spermogram is normal. This detailed analysis on the levels of the various markers can also aid in the selection of the most efficient treatment of these infertilities and in obtaining better results while performing ART. Zinc and several other anti-oxidants are being currently used as supplements for treating male infertility [43,54,55,56]. Supplementation of the medium with some analytes found in this study may improve efficiency while performing ART. Additionally, the above-discussed markers may also be used as a tool for selecting a sperm donor for IVF, ensuring a successful outcome—a major issue during IVF.

## 5. Limitations of the Study

This study represents a preliminary analysis; it primarily consists of local communities and is not a population-based study design. Thus, large, diverse cohorts are required to confirm the associations between these markers. Therefore, the firm conclusions about how these are relevant to the general population must be made with caution. Additionally, the results were obtained in a relatively limited number of male infertility profiles in accordance with regionally observed incidences. More sub-categories of infertile patients (such as oligoasthenozoospermia, teratozoospermia, teratooligozoospermia, etc.) need to be studied to understand the definite effect of these biochemical and immunological variations on male infertility. Therefore, we further suggest investigating the markers in larger cohorts and subgroups.

## 6. Conclusions

To our knowledge, this study is the first to provide initial evidence to support the use of human seminal plasma in the diagnosis of male infertility wherein a wide range of biochemical and immunological markers have been analyzed on a single patient. The results, which try to correlate various components with different infertility associations, indicate that the present dataset could serve as a resource for exploring key markers for male infertility and might be of clinical interest in the infertility investigation. Further studies are required to reinforce this by increasing the sample size and by determining the efficacy of specific treatments on the biochemical and immunological quality of seminal plasma.

## Figures and Tables

**Table 1:** Comparison of age, sperm count, and motility between fertile control and various infertile groups. Age and sperm parameters are expressed as Mean ± SD and one-way ANOVA is used to calculate the *p*-value.

Parameter	Study Groups					*p* Value
	FC (*n* = 50)	AS (*n* = 13)	NOA (*n* = 16)	NI (*n* = 10)	OZ (*n* = 11)	
**AGE** **(Years)**	27.94 ± 2.34	30.61 ± 2.90	29.75 ± 3.15	29.3 ± 2.45	30.18 ± 3.57	0.0056
**Sperm Count** **(Per Million)**	70.32 ± 16.90	44.92 ± 12.46	0	59.50 ± 8.32	7.09 ± 2.30	<0.0001
**Sperm Motility** **(Percentage)**	65.96 ± 14.11	7.00 ± 1.83	0	54.40 ± 8.54	53.73 ± 10.39	<0.0001

FC: fertile control; AS: asthenozoospermia; NOA: non-obstructive azoospermia; NI: normozoospermia infertile; OZ: oligozoospermia.

**Table 2:** Comparison of the 21 different biochemical and immunological markers observed between fertile control and various infertile groups. The values are expressed as Mean ± SD and one-way ANCOVA is used to calculate the *p*-value.

Markers	FC	AS	NOA	NI	OZ	*p* Value
**Analytes**						
**GLC (mg/dL)**	8.972 ± 0.859	4.337 ± 1.666	3.430 ± 1.475	9.643 ± 1.855	2.704 ± 1.788	0.0001
**TG (mg/dL)**	20.344 ± 2.221	26.711 ± 4.294	30.522 ± 3.900	50.539 ± 4.770	33.241 ± 4.602	0.0001
**CHOL (mg/dL)**	32.353 ± 2.312	49.100 ± 4.485	37.724 ± 3.972	42.289 ± 4.993	49.506 ± 4.812	0.003
**UA (mg/dL)**	3.374 ± 0.310	4.265 ± 0.601	3.326 ± 0.533	3.391 ± 0.669	5.267 ± 0.645	0.089
**Urea (mg/dL)**	126.967 ± 7.269	135.625 ± 14.099	112.945 ± 12.485	80.598 ± 15.694	105.139 ± 15.126	0.052
**miP (mg/dL)**	763.987 ± 21.739	886.422 ± 42.166	657.509 ± 37.340	595.456 ± 46.938	758.115 ± 45.238	<0.0001
**TP (g/dL)**	2.761 ± 0.128	2.661 ± 0.248	2.631 ± 0.219	2.218 ± 0.276	2.819 ± 0.266	0.475
**ALB (mg/dL)**	0.920 ± 0.063	1.110 ± 0.122	0.867 ± 0.108	0.712 ± 0.136	0.890 ± 0.131	0.285
**CREAT (mg/dL)**	5.761 ± 0.289	4.595 ± 0.560	5.454 ± 0.496	4.628 ± 0.624	4.819 ± 0.601	0.239
**Enzymes**						
**SGOT (U/L)**	18.676 ± 1.331	11.548 ± 2.582	16.063 ± 2.286	6.692 ± 2.874	10.129 ± 2.77	0.001
**SGPT (U/L)**	54.363 ± 2.962	118.526 ± 5.744	65.562 ± 5.087	30.511 ± 6.395	48.063 ± 6.163	0.0001
**CK (U/L)**	291.549 ± 26.037	413.875 ± 50.504	111.383 ± 44.724	248.598 ± 56.22	147.689 ± 54.183	<0.0001
**LDH (U/L)**	30.968 ± 2.7	4.489 ± 5.237	20.654 ± 4.638	11.462 ± 5.83	15.176 ± 5.619	<0.0001
**Trace Elements**						
**CAL (mmol/L)**	4.474 ± 0.058	4.431 ± 0.113	4.346 ± 0.1	4.535 ± 0.126	4.415 ± 0.121	0.779
**Zinc (mg/L)**	134.238 ± 4.848	62.678 ± 9.403	63.852 ± 8.327	137.731 ± 10.467	74.671 ± 10.088	<0.0001
**Sodium (mmol/L)**	107.300 ± 2.103	96.178 ± 4.08	103.938 ± 3.613	101.973 ± 4.542	125.995 ± 4.377	<0.0001
**Immunological Markers**						
**WBC (10^3^/mm^3^)**	0.042 ± 0.03	0	0.580 ± 0.052	0	0.42 ± 0.028	<0.001
**LYM (%)**	0	0	55.575 ± 1.983	0	0	-
**MON (%)**	0	0	7.395 ± 0.234	0	0	-
**GRAN (%)**	0	0	28.825 ± 1.345	0	0	-
**PLT (10^3^/mm^3^)**	7.006 ± 1.229	30.585 ± 2.384	12.199 ± 2.111	10.978 ± 2.653	7.921 ± 2.557	<0.0001

GLC: glucose; TG: triglycerides; CHOL: cholesterol; UA: uric acid; miP: microprotein; TP: total protein; ALB: albumin; CREAT: creatinine; SGPT: serum glutamic pyruvic transaminase; SGOT: serum glutamic oxaloacetic transaminase; CK: creatine kinase; LDH: lactate dehydrogenase; CAL: calcium; WBC: white blood cells; LYM: lymphocytes; MON: monocytes; GRA: granulocytes; PLT: platelets.

**Table 3:** Pathway analysis with possible hits and impacts using MetaboAnalyst 4.0. **Total compounds** represent the total number of compounds in the pathway; **Hits** is the actually matched number from the user uploaded data; **Raw p** is the original *p*-value calculated from the enrichment analysis; **Holm p** is the *p*-value adjusted by Holm–Bonferroni method; **FDR** is the false discovery rate adjusted *p*-value; **Impact** is the pathway impact value calculated from pathway topology analysis.

Pathway	Total Compounds	Hits	Raw p	-log(10)p	Holm p	FDR	Impact
**Primary bile acid biosynthesis**	46	1	3.04 × 10^−4^	3.52	2.43 × 10^−3^	8.10 × 10^−4^	0.05
**Steroid biosynthesis**	42	1	3.04 × 10^−4^	3.52	2.43 × 10^−3^	8.10 × 10^−4^	0.03
**Steroid hormone biosynthesis**	85	1	3.04 × 10^−4^	3.52	2.43 × 10^−3^	8.10 × 10^−4^	0.01
**Glycogen metabolism**	18	1	1.78 × 10^−3^	2.75	8.90 × 10^−3^	2.37 × 10^−3^	0.42
**Galactose metabolism**	27	1	1.78 × 10^−3^	2.75	8.90 × 10^−3^	2.37 × 10^−3^	0.03
**Neomycin, kanamycin, and gentamicin biosynthesis**	2	1	1.78 × 10^−3^	2.75	8.90 × 10^−3^	2.37 × 10^−3^	0.00
**Purine metabolism**	65	2	1.14 × 10^−1^	9.43 × 10^−1^	2.28 × 10^−1^	1.14 × 10^−1^	0.00
**Arginine biosynthesis**	14	1	1.14 × 10^−1^	9.43 × 10^−1^	2.28 × 10^−1^	1.14 × 10^−1^	0.00

**Table 4:** Tabulated representation of only those biochemical and immunological markers whose levels were significantly altered in the various infertile subgroups as compared to the fertile controls. S = significant and (-) represents non-significant value. Upward and downward arrows represent the increased or decreased levels of the marker, respectively.

Seminal Plasma Markers	Infertility Groups			
	AS	NOA	NI	OZ
**GLC**	-	S↓	-	S↓
**TG**		-	S↑	S↑
**CHOL**	S↑	-	-	S↑
**miP**	-	-	S↓	-
**SGPT**	S↑	-	S↓	-
**SGOT**	-	-	S↓	-
**CK**	-	S↓	-	-
**LDH**	S↓	-	S↓	-
**Zinc**	S↓	S↓	-	S↓
**Sodium**	-	-	-	S↑
**WBC**	-	S↑	-	-
**PLT**	S↑	-	-	-

GLC: glucose; TG: total glycerides; CHOL: cholesterol; miP: micro-protein; SGPT: serum glutamic pyruvic transaminase; SGOT: serum glutamic oxaloacetic transaminase; CK: creatine kinase; LDH: lactate dehydrogenase; WBC: white blood cells; PLT: platelets.

**Table 5:** The correlation between the biochemical markers, immunological markers, and sperm parameters within the fertile control and infertile group. *p* < 0.01 was taken as the statistical significance limit.

Variable 1	Variable 2	Correlation (r)	Lower 95%	Upper 95%	Probability (P)
**Fertile Controls**					
ALB	TP	0.5796	0.3591	0.7387	<0.0001
TP	CHOL	0.4878	0.2423	0.6746	0.0003
CK	TP	0.4469	0.1926	0.6450	0.0011
CK	UREA	0.3967	0.1331	0.6079	0.0043
CK	TG	0.3977	0.1312	0.6106	0.0047
UREA	TP	0.3679	0.0998	0.5862	0.0086
CAL	SGOT	−0.3604	−0.5805	−0.0912	0.0101
% Motility	CAL	−0.3523	−0.5743	−0.0820	0.0121
**Infertile Patients**					
MON	LYM	0.9539	0.9203	0.9739	<0.0001
GRA	MON	0.9039	0.8358	0.9446	<0.0001
GRA	LYM	0.8244	0.7086	0.8969	<0.0001
PLT	CK	0.6622	0.4706	0.7942	<0.0001
TG	GLC	0.6665	0.4742	0.7981	<0.0001
Cell count	CK	0.6554	0.4612	0.7897	<0.0001
Cell count	MON	−0.6266	−0.7706	−0.4219	<0.0001
SGOT	UREA	0.6217	0.4152	0.7673	<0.0001
SGPT	UREA	0.6213	0.4147	0.7670	<0.0001
Cell count	LYM	−0.6178	−0.7647	−0.4099	<0.0001
% Motility	MON	−0.6060	−0.7567	−0.3941	<0.0001
% Motility	LYM	−0.5974	−0.7509	−0.3827	<0.0001
Cell count	GRA	−0.5863	−0.7434	−0.3681	<0.0001
miP	TG	−0.5859	−0.7444	−0.3648	<0.0001
miP	SGPT	0.5756	0.3540	0.7360	<0.0001
miP	UREA	0.5736	0.3513	0.7346	<0.0001
% Motility	GRA	−0.5670	−0.7301	−0.3428	<0.0001
CK	SGPT	0.5533	0.3251	0.7207	<0.0001
% Motility	SGPT	−0.5530	−0.7204	−0.3246	<0.0001
PLT	SGPT	0.5437	0.3127	0.7140	<0.0001
miP	ALB	0.5372	0.3044	0.7095	<0.0001
LYM	CK	−0.5334	−0.7068	0.2995	<0.0001
MON	CK	−0.5255	−0.7013	−0.2895	<0.0001
% Motility	Zinc	0.5219	0.2849	0.6987	0.0001
LYM	LDH	0.5131	0.2739	0.6925	0.0001

## Data Availability

The data that support the findings of this study are not publicly available. However, data are available from the authors upon reasonable request.

## Supplementary Materials

The following are available online at https://www.mdpi.com/article/10.3390/cimb43030093/s1, Table S1: MetaboAnalyst 4.0 analysis of compound name mapping. The data integrity check was performed to ensure that the uploaded compounds meet the basic requirements such as a valid HMDB ID, PubChem number, and KEGG ID for meaningful downstream analysis.
