# Supplementary material for: A Comparative Analysis of the Altered Levels of Human Seminal Plasma Constituents as Contributing Factors in Different Types of Male Infertility
Source: Curr Issues Mol Biol. 2021 Sep 26;43(3):1307–24. doi: 10.3390/cimb43030093 (PMC8929149; doi:10.3390/cimb43030093)
Supplement: Supplementary file 1 [file cimb-43-00093-s001.zip › cimb-1347614-supplementary.pdf]

Supplementary Table S1: MetaboAnalyst 4.0 analysis of compound name mapping. The data integrity check was performed to ensure that the uploaded compounds meet the basic requirements such as a valid HMDB ID, PubChem number, and KEGG ID for meaningful downstream analysis.

Query	Match	HMDB	PubChem	KEGG
**Glucose**	D-Glucose	HMDB0000122	5793	C00031
**Cholesterol**	Cholesterol	HMDB0000067	5997	C00187
**Zinc**	Zinc	HMDB0015532	32051	C00038
**Sodium**	Sodium	HMDB0000588	923	C01330
